# Mini Review on the Use of Clinical Cancer Registers for Prostate Cancer: The National Prostate Cancer Register (NPCR) of Sweden

**DOI:** 10.3389/fmed.2019.00051

**Published:** 2019-03-22

**Authors:** Walter Cazzaniga, Eugenio Ventimiglia, Massimo Alfano, David Robinson, Ingela Franck Lissbrant, Stefan Carlsson, Johan Styrke, Francesco Montorsi, Andrea Salonia, Pär Stattin

**Affiliations:** ^1^Division of Experimental Oncology/Unit of Urology, URI, IRCCS Ospedale San Raffaele, Milan, Italy; ^2^University Vita-Salute San Raffaele, Milan, Italy; ^3^Department of Urology, Ryhov Hospital, Jonköping, Sweden; ^4^Institute of Clinical Sciences, Department of Oncology, Sahlgrenska Academy, University of Göteborg, Göteborg, Sweden; ^5^Division of Urology, Karolinska University Hospital, Stockholm, Sweden; ^6^Department of Molecular Medicine and Surgery (MMK), Karolinska Institutet, Stockholm, Sweden; ^7^Department of Surgical and Perioperative Sciences, Urology and Andrology, Umeå University, Umeå, Sweden; ^8^Department of Surgical Sciences, Uppsala University, Uppsala, Sweden

**Keywords:** National Prostate Cancer Register (NPCR) of Sweden, Prostate Cancer data Base Sweden (PCBaSe), clinical cancer register, prostate cancer, online registration, report

## Abstract

Given the increasing prevalence of cancer, it is vital to systematically collect data in order to monitor disease trends and quality of cancer care. For this purpose, clinical cancer registries have been developed in some countries. These registers are intended to be used as a basis for quality assurance and quality improvement, but they also constitute a rich resource of real world data for research. The aim of this mini-review was to describe the structure and the organization of the National Prostate Cancer Register (NPCR) with some examples on how data in NPCR have affected prostate cancer care in Sweden.

## Introduction

Prostate cancer (Pca) is the most common cancer in many Western countries. In 2017 there were estimated to be 161,360 incident cases of Pca and 26,730 Pca related deaths in the United States, making it the most frequently diagnosed neoplasm in men and the third leading cause of male cancer death (1). Pca is a multifaceted disease: it can be indolent and asymptomatic as well as aggressive with a poor prognosis, with a wide range of treatments strategies (1).

Given the high incidence and prevalence of Pca it is vital to develop systematic and extensive data collection in order to address several clinical issues, with the ultimate goal to improve both clinical practice and health policies (2, 3), along with patients well-being.

Clinical cancer registries (also known as quality registers) have been developed in some countries, especially in northern Europe. For instance, there are more than 20 clinical cancer registries in Sweden and in total there are over 100 quality registers for various diseases and surgical procedures (4). The purpose of these registers is to collect data for quality assurance and quality improvement in health care (5). To obtain these goals, a central administration needs to be created with the specific aim to coordinate various collaborations. Some of the clinical registers have had a major impact on clinical practice. For example, the extremely low revision rate of hip implants in Sweden has been attributed to the systematic feedback on different implants' failure rate reported in the Swedish Hip Arthroplasty Register (6). In addition to quality assurance and care improvement, these registers also represent a very rich source of real world data for research.

The aim of this review is to describe the structure and organization of the National Prostate Cancer Register (NPCR), the largest clinical cancer register in Sweden that comprise more than 180,000 Pca cases with data regarding several aspect of both the disease and the socioeconomical condition of those men. Moreover, we demonstrate how the implementation of NPCR has affected some specific aspects of clinical practice for Pca in Sweden.

## The National Prostate Cancer Register of Sweden

NPCR registers comprehensive data on cancer characteristics, diagnostic work-up, and primary treatment for patients diagnosed with Pca in Sweden (7). Since 1998, NPCR includes information for 98% of all incident Pca cases registered in the Swedish Cancer Registry, to which reporting is compulsory and mandated by law (8).

The steering group of NPCR consists of a register holder, one urologist and one oncologist from each of the six health care regions of Sweden and in addition, as well as a register nurse, a register coordinator, a dedicated uro-pathologist, a biostatistician, an epidemiologist and two Pca patient representatives.

Data entry and registration for Pca in NPCR is performed by dedicated staff at each department by use of four online-forms: a diagnostic form, a form for work-up and medical treatment, and separate forms for radiotherapy (RT) since 2007 and radical prostatectomy (RP) since 2015. Specifically, Pca pathological classification at diagnosis is registered according to the Gleason classification; furthermore, more detailed data regarding diagnostic biopsies (e.g., total number of biopsy cores, number of positive cores, and so on) are registered too. Recent analyses of data quality in NPCR, found that data were virtually complete, representative, and had overall high quality (9–11). A list of the complete collected variables (in Swedish), including capture and descriptive statistics at various levels (e.g., region, county) can be found at www.npcr.se (English version, http://npcr.se/in-english/).

**Figure 1:**
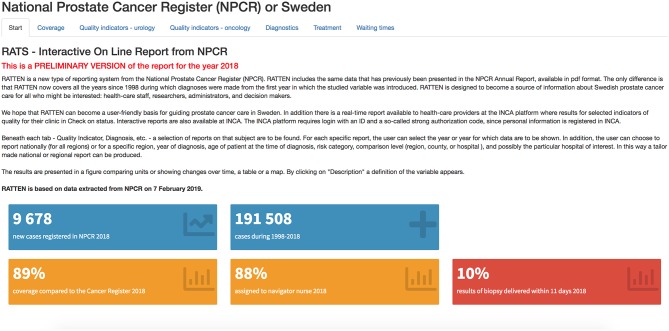
Screenshot of RATTEN, a reader-friendly format for accessing information in NPCR (preliminary data for 2018).

Annually, a report presenting information at a department level is publicly available at www.npcr.se. This report includes key data on quality aspects of the register itself as well as characteristics of cancer cases. In addition, there is an online real time report presenting data on ten quality indicators for Pca care selected from the National Prostate Cancer Guidelines available at the INCA platform with secured access for staff involved in direct patient care (12). This report provides a dashboard panel with an *at-a-glance* feedback to health care providers on their performance. In 2016, a public online interactive report was created and posted on www.npcr.se/RATTEN (13) and this now provides the most reader-friendly format for information in NPCR (Figure 1).

## Prostate Cancer Data Base Sweden (PCBaSe)

**Figure 2:**
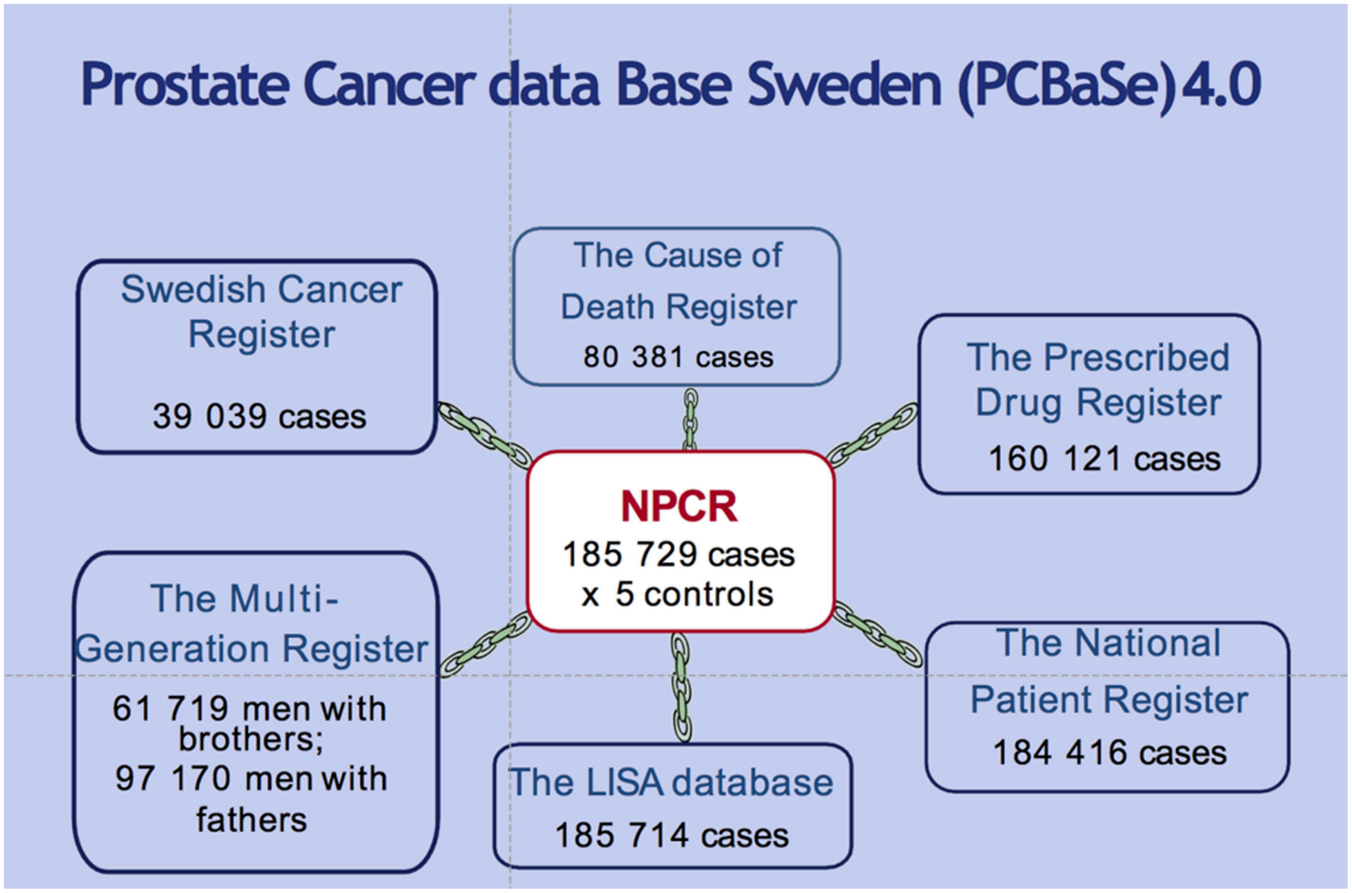
Linkages and number of cross-linked men in the Prostate Cancer Database Sweden (PCBaSe) 4.0. The inclusion criteria for NPCR are applied for all those sub registries.

In 2008, the NPCR was linked to a number of other population-based health-care registers and demographic databases to create the Prostate Cancer Database Sweden (PCBaSe), a platform for clinical research (Figure 2). The linkage was performed with the Swedish Cancer Registry, the Cause of Death Register, the Prescribed Drug Registry, the National Patient Registry, the Longitudinal Integration Database for Health Insurance and Labor Market Studies (LISA) and the Multi-Generation Registry. This process was made possible by the use of a personal identity number, available for all Swedish citizens (14). PCBaSe also includes five prostate cancer-free control men for each Pca case, randomly selected from groups of men matched to the respective Pca case on birth year and county of residence. The addition of these controls allows for case–control studies as well as for cohort comparisons following cases and controls after date of diagnosis.

### The Cause of Death Registry

Since 1991, the Cause of Death Registry collects the cause of death for all persons registered in Sweden with the use of the ICD codes (15, 16). The validity of the registered data in this registry has been found to be high, 86% overall agreement with reviewed medical records (17).

### The Prescribed Drug Registry

The Prescribed Drug Registry comprises all out-patient filled prescriptions in Sweden since July 2005 with data on drug (recorded with ATC codes), daily dose and date of prescription (18). It is extensively used for research purpose.

### The National Patient Registry

The National Patient Registry includes information regarding all in-patient and out-patient care in Sweden from 1987. The registry is updated monthly by each Swedish county and contains data on performed procedures; moreover, the hospital and the dates of the in-patients stay are detailed. This register is used to double check the data collected via NPCR forms and for calculating a comorbidity index (Charlson Comorbidity Index; CCI) (19). Recent studies evaluating the validity of the National Patient Register confirmed a value between 85 and 95% for different diseases (20).

### The Longitudinal Integration Database for Health Insurance and Labor Market Studies (LISA)

The Longitudinal Integration Database for Health Insurance and Labor Market Studies (LISA) is a nation-wide register which aims to collect data regarding educational level, income, civil status and type of employment for Swedish individuals aged 16 onwards. The availability of these variables in PCBaSe allows to investigate socio-economic factors associated with treatment outcomes and efficacy (21).

### Multi-Generation Registry

The Multi-Generation Registry includes information on the family for every subject born in Sweden since 1932 and who was still a resident in Sweden from 1961 onwards (22). As for PCBaSe, the index case was defined as a man with Pca, registered in NPCR, with at least one brother identified in the Multi-Generation Registry.

Since its inception in 2009, PCBaSe has been the basis for over 100 peer-reviewed articles on a wide range of topics, including health-care patterns, outcome studies and post-authorization safety studies (PASS) of rare adverse events of drug treatment.

## Effects of NPCR on Quality of Prostate Cancer Care in Sweden

### Changes in Pre-operative Work-Up for Low-Risk Pca

Due to the introduction of the PSA testing as a screening tool for Pca, there has been a strong shift toward low-risk disease, making an extensive imaging investigation unnecessary at diagnosis (23). In particular, different professional societies have focused their attention on the reduction of the use of the whole-body radionuclide bone scan, since the detection rate of metastases is extremely low in low-risk patients compared to the potential false positive rate of this technique (24). The NPCR records and regularly reports the proportion of men with low-risk Pca who undergo a bone scan at each department, in order to decrease the use of unnecessary investigations and promoting adherence to guidelines. A recent study analyzed changes over time of the proportion of men with low-risk Pca who underwent bone scan in Sweden using data from NPCR. The results showed a decrease from 45% in 1998 to 3% in 2008 and 2009 of bone scan among low risk Pca men (25). Although the retrospective study design does not allow to demonstrate causality between the decline in inappropriate Pca imaging in Sweden and the NPCR's report, this represent an example of big data applied to real life setting with the aim to improve the quality of care for Pca men.

### Increased Use of Local Treatment for Locally Advanced Pca

Currently the European Association of Urology (EAU) Guidelines recommend, for locally advance Pca, the use of a combination of androgen deprivation therapy (ADT) and radiotherapy (RT). This recommendation is based on the results of randomized controlled trials, such as the SPCG-7 that demonstrated a decrease in Pca mortality in men with locally advanced Pca if treated with ADT + RT compared to ADT alone (26). In Sweden, among healthy men aged 70–80 years with high-risk non-metastatic Pca [defined as Pca with no evidence of metastasis (N0 or Nx, M0 or Mx) and at least one of the following three criteria: Gleason score 8–10, local clinical stage T3, or prostate-specific antigen (PSA) 20–49 ng/ml], there has been a significant increase of use of curative treatment, up from 10% in 2001 to almost 50% in 2012 (27). At the same time, an observational study reported a higher Pca-specific mortality for men harboring locally advanced Pca and managed with non-curative intent, suggesting a role for a more active treatment (28). Also in this case, the reports of NPCR may have affected clinical practice (12).

### Increased Use of Active Surveillance for Low-Risk Prostate Cancer

In 2007, the Swedish guidelines for Pca care recommended active surveillance for patients with low-risk Pca. Both this recommendation and the real-time feedback to departments on their adherence to the national guidelines by NPCR have potentially contributed to the increasing uptake of active surveillance in Sweden (29). In fact, it has been subsequently reported a steep increase in the adoption of this treatment strategy among men with low-risk Pca, up from 40% in 2009 to 74% in 2014 (29). Even more active surveillance is used for very-low risk Pca from 57% in 2009 to 91% in 2014.

## Conclusion

To date, prostate cancer represents a major health concern for men, due to its high incidence and prevalence. For this reason, there is a need for data that can be used as metrics for quality assurance, improvement, benchmarking and clinical research with the final aim to improve Pca care. In this review, we have shown some examples how NPCR affected Pca care in Sweden. In the future, additional analysis of NPCR data, such those regarding Patients Reporting Outcomes Measures (PROMs) will help to further improve the quality of prostate cancer care.

## Author Contributions

WC and EV drafted the manuscript. All authors revised the manuscript and approved the final version to be published.

### Conflict of Interest Statement

The authors declare that the research was conducted in the absence of any commercial or financial relationships that could be construed as a potential conflict of interest.
